# Supplementary material for: Magnetothermal genetic deep brain stimulation of motor behaviors in awake, freely moving mice
Source: eLife. 2017 Aug 15;6:e27069. doi: 10.7554/eLife.27069 (PMC5779110; doi:10.7554/eLife.27069)
Supplement: Transparent reporting form [file elife-27069-transrepform.pdf]

eLIFE

1st Floor
24 Hills Road
Cambridge CB2 1JP, UK

P 01223 855340
W elifesciences.org
T @elife

eLife's transparent reporting form

We encourage authors to provide detailed information *within their submission* to facilitate the interpretation and replication of experiments. If you have any questions, please contact us: editorial@elifesciences.org.

Sample-size estimation

- You should state whether an appropriate sample size was computed when the study was being designed
- You should state the statistical method of sample size computation and any required assumptions
- If no explicit power analysis was used, you should describe how you decided what sample (replicate) size (number) to use

Please outline where this information can be found within the submission (e.g., page numbers or figure legends), or explain why this information doesn't apply to your submission:

When designing the in-vivo stimulation experiments, a power analysis using G-power was performed. These experiments mostly replicate optogenetics experiments, so the effect size can be predicted. For an effect size of 3, stimulation leading to ambulation, G-power calculates that 4 animals in each group would provide a confidence larger than 98% for one-tail (comparing means of two independent groups). Hence, we used at least four animals in each group as biological replicator; the technical replication was higher.

For the stimulation of TRPV1+ / NP hippocampal neurons in culture we did not perform an a-priory power analysis because we did not know the expected effect size, the in-vitro work is supporting the in-vivo but wasn't the main focus as it's not novel, and because each culture provides a large number of neurons. On page 10 we state that we recorded 79 neurons from six separate cultures to account for biological and technical variations, with 87% spiking within the first 5s of stimulation.

Replicates

- You should report how often each experiment was performed
- You should include a definition of biological versus technical replication
- The data obtained should be provided and sufficient information should be provided to indicate the number of independent biological and/or technical replicates
- If you encountered any outliers, you should describe how these were handled
- Criteria for exclusion/inclusion of data should be clearly stated
- High-throughput sequence data should be uploaded before submission, with a private link for reviewers provided (these are available from both GEO and ArrayExpress)

Please outline where this information can be found within the submission (e.g., page numbers or figure legends), or explain why this information doesn't apply to your submission:

eLife Sciences Publications, Ltd is a limited liability non-profit non-stock corporation incorporated in the State of Delaware, USA, with company number 5030732, and is registered in the UK with company number FC030576 and branch number BR015634 at the address 1st Floor, 24 Hills Road, Cambridge CB2 1JP | August 2014

Biological replications were virus and nanoparticle injections in distinct mice with subsequent field stimulation. In the manuscript, these are counted as mice in control of experimental group.

Technical replications are repeated applications of the alternating magnetic field to the same animal. This was done in two forms: as repeated (3x - 4x) one-minute application over the course of a 15-20 minute experiments, and be repeating this entire sequence on the same animal 24h and 48hrs later. These numbers are reported throughout the manuscript as number of stimulations (pages 11-14, figures 4-6).

No outliers were encounter. All data points are in the figure as scatter plots. Only two animals were removed from the study: one which got too agitated when placed into the experiment arena and one which did not recover well from stereotactic surgery

Statistical reporting

- Statistical analysis methods should be described and justified
- Raw data should be presented in figures whenever informative to do so (typically when N per group is less than 10)
- For each experiment, you should identify the statistical tests used, exact values of N, definitions of center, methods of multiple test correction, and dispersion and precision measures (e.g., mean, median, SD, SEM, confidence intervals; and, for the major substantive results, a measure of effect size (e.g., Pearson's r, Cohen's d)
- Report exact p-values wherever possible alongside the summary statistics and 95% confidence intervals. These should be reported for all key questions and not only when the p-value is less than 0.05.

Please outline where this information can be found within the submission (e.g., page numbers or figure legends), or explain why this information doesn't apply to your submission:

All experiments compare two conditions, and hence ANOVA t-test was used to determine significance (p-values). Means, SEM, number of samples, and significance are reported in each figure and in the results section.

For the in-vivo experiments were N are between 4 and 16, all raw data is shown in scatter plots (figures 4-6), whereby it's clearly marked if N is a biological replication (individual animals) or a technical replication (trials).

(For large datasets, or papers with a very large number of statistical tests, you may upload a single table file with tests, Ns, etc., with reference to page numbers in the manuscript.)

eLife Sciences Publications, Ltd is a limited liability non-profit non-stock corporation incorporated in the State of Delaware, USA, with company number 5030732, and is registered in the UK with company number FC030576 and branch number BR015634 at the address 1st Floor, 24 Hills Road, Cambridge CB2 1JP | August 2014

Additional data files ("source data")

- We encourage you to upload relevant additional data files, such as numerical data that are represented as a graph in a figure, or as a summary table
- Where provided, these should be in the most useful format, and they can be uploaded as "Source data" files linked to a main figure or table
- Include model definition files including the full list of parameters used
- Include code used for data analysis (e.g., R, MatLab)
- Avoid stating that data files are "available upon request"

Please indicate the figures or tables for which source data files have been provided:

We have included for each in-vivo experiment one data set in form of a recorded video. That is the direct raw data. We refrain from including all the intermediate steps, such as tracked X,Y position or speed, as that is derived from the video data.
